# Cyclin-dependent kinase 5 stabilizes hypoxia-inducible factor-1α: a novel approach for inhibiting angiogenesis in hepatocellular carcinoma

**DOI:** 10.18632/oncotarget.8342

**Published:** 2016-03-24

**Authors:** Julia Herzog, Sandra M. Ehrlich, Lisa Pfitzer, Johanna Liebl, Thomas Fröhlich, Georg J. Arnold, Wolfgang Mikulits, Christine Haider, Angelika M. Vollmar, Stefan Zahler

**Affiliations:** ^1^ Department of Pharmacy, Pharmaceutical Biology, University of Munich, Munich, Germany; ^2^ Laboratory for Functional Genome Analysis (LAFUGA), Gene Center Munich, University of Munich, Munich, Germany; ^3^ Department of Medicine I, Institute of Cancer Research, Comprehensive Cancer Center Vienna, Medical University of Vienna, Vienna, Austria

**Keywords:** angiogenesis, HIF-1α, CDK5, HCC

## Abstract

We recently introduced CDK5 as target in HCC, regulating DNA damage response. Based on this and on our previous knowledge about vascular effects of CDK5, we investigated the role of CDK5 in angiogenesis in HCC, one of the most vascularized tumors. We put a special focus on the transcription factor HIF-1α, a master regulator of tumor angiogenesis.

The interaction of CDK5 with HIF-1α was tested by Western blot, PCR, reporter gene assay, immunohistochemistry, kinase assay, co-immunoprecipitation, mass spectrometry, and mutation studies. *In vivo*, different murine HCC models, were either induced by diethylnitrosamine or subcutaneous injection of HUH7 or HepG2 cells. The correlation of vascular density and CDK5 was assessed by immunostaining of a microarray of liver tissues from HCC patients.

Inhibition of CDK5 in endothelial or HCC cells reduced HIF-1α levels *in vitro* and *in vivo*, and transcription of HIF-1α target genes (*VEGFA*, *VEGFR1*, *EphrinA1*). Mass spectrometry and site directed mutagenesis revealed a stabilizing phosphorylation of HIF-1α at Ser687 by CDK5. Vascular density was decreased in murine HCC models by CDK5 inhibition.

In conclusion, inhibiting CDK5 is a multi-modal systemic approach to treat HCC, hitting angiogenesis, as well as the tumor cells themselves.

## INTRODUCTION

CDK5, a serine/threonine kinase, has been identified more than 20 years ago in bovine brain extract [1]. It is unique among CDKs because it is no classical mediator of cell-cycle transitions, but is implicated in various aspects of neuronal development, function and disease. In recent years extra-neuronal functions of CDK5 have become increasingly clear [2]: it promotes cell migration in pancreatic and lung cancer [3, 4], and is involved in medullary thyroid carcinoma progression [5]. Recently, we have reported that CDK5 is highly expressed in hepatocellular carcinomas, and regulates DNA damage responses in HCC cells [6].

As a contribution to the newly emerging field of extra-neuronal roles of CDK5, we have demonstrated surprising effects in the vascular context: 1) in endothelial cells, CDK5 is important for migration and angiogenesis by regulating the small GTPase Rac-1 [7], 2) it regulates inflammatory responses of the endothelium by influencing the transcription factor NFkappaB [8], and 3) it directly phosphorylates the transcription factor FOXC2, thus steering lymphatic development [9].

The induction of angiogenesis is regarded as one of the hallmarks of cancer [10], since the establishment of new blood vessels from pre-existing ones provides growing tumors with oxygen and nutrients. This promotes progression and invasion, as well as the formation of metastasis [11]. Hepatocellular carcinoma (HCC) is one of the most vascularized solid tumors [12], showing increased vascular endothelial growth factor (VEGF) expression [13], directly correlating with worse overall survival in patients [14]. Since to date no satisfying systemic treatment against HCC exists, anti-angiogenic therapy has become of increasing interest in clinical management of HCC [15].

The most prominent anti-angiogenic drugs currently used for cancer therapy in general, such as the antibody bevacizumab [11] or the multikinase inhibitors sorafenib and sunitinib, target VEGF signaling [16]. However, patients inescapably develop resistances [17] by upregulation of other growth factors [18], or the induction of hypoxia inducible factors (HIFs) [17, 19], central mediators of angiogenesis [20]. A further pitfall of anti-angiogenic therapy seems to be that the resulting tumor hypoxia causes an immunosuppressed and prometastatic niche [21]. New therapeutic options, which could target such evasive mechanisms, and finally lead to a higher efficacy of anti-angiogenic therapy, are still lacking.

We investigated whether CDK5, in addition to its direct effects on HCC cells, also has anti-angiogenic effects in HCC, and identified the underlying mechanism: we demonstrate that CDK5 stabilizes HIF-1α, a master regulator of angiogenesis, by phosphorylation at serine 687 in endothelial and liver cancer cells, and proof the potential of CDK5 as a new target for anti-angiogenic HCC therapeutics by reducing vascularization in tumors of different murine HCC models through pharmacological or genetic inhibition of CDK5.

## RESULTS

### Pharmacological inhibition of CDK5 inhibits angiogenesis *in vivo* and expression of CDK5 correlates with vascular density in a human tissue microarray

**Figure 1:**
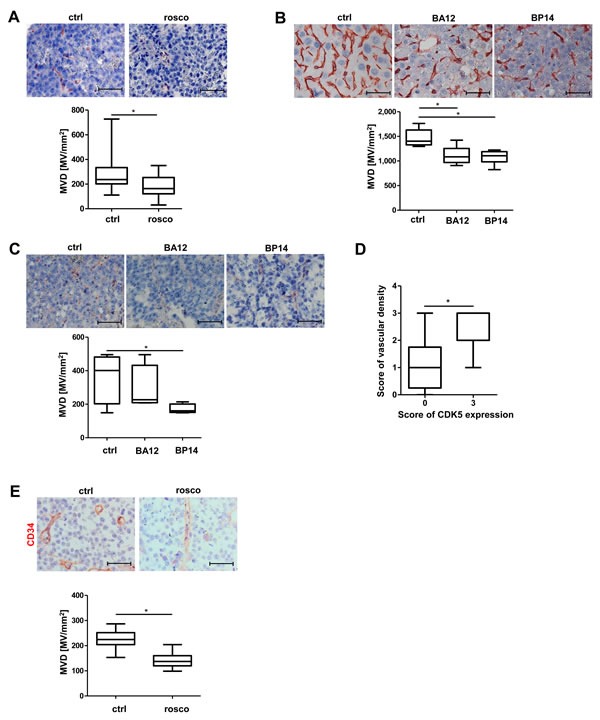
Pharmacological inhibition of CDK5 reduces vascular density in different murine liver tumor models **A.** Immunostaining for CD31 (red) and haematoxylin (blue, nuclei) of HCC tumors grown in SCID mice either treated with solvent or roscovitine. Microvessel density (MVD) was determined. Non-parametric t-test on Mann-Whitney, * P < .05, *n* = 8. Whisker lines indicate maximum and minimum values. **B.** Immunostaining for CD31 (red) and haematoxylin (blue, nuclei) of DEN-induced tumors grown in mice either treated with solvents or the roscovitine derivatives BA12 and BP14. MVD was determined. Non-parametric t-test on Mann-Whitney, *P < .05, *n* = 5. Whisker lines indicate maximum and minimum values. **C.** Immunostaining for CD31 (red) and haematoxylin (blue, nuclei) of Hep G2 tumors either treated with solvents or the roscovitine derivatives BA12 and BP14. MVD was determined. Non-parametric t-test on Mann-Whitney, *P < .05, *n* = 4. Whisker lines indicate maximum and minimum values. **D.** Immunostaining of a human HCC tissue microarray for CD31. Staining was assessed by the following score: 0 – absent, 1 – weak, 2 – moderate, 3 – strong expression. Non-parametric t-test on Mann-Whitney, *P < .05, *n* = 12 (CDK5 score 0), *n* = 8 (CDK5 score 3). Whisker lines indicate maximum and minimum values. Scale bars: 50 μm. **E.** Immunostaining for CD34 (red) and haematoxylin (blue, nuclei) of HCC tumors grown in SCID mice either treated with solvent or roscovitine is shown. The microvessel density (MVD) per square mm was determined. Scale bars: 50 μm. Non-parametric t-test on Mann-Whitney, *P < .05, *n* = 8. Whisker lines indicate maximum and minimum values.

We have previously shown that treatment of HUH7 tumors with roscovitine (rosco), a well-established CDK5 inhibitor [22], or downregulation of CDK5 by siRNA in HCC cells, causes reduction of tumor growth to about one half or less [6]. Vessel staining for CD31showed reduced microvascular density in these tumors (Figure 1A). This result was confirmed by CD34 staining, another endothelial cell marker, additionally expressed on hematopoietic stem cells [23] (Figure 1E). Treatment of mice with the novel roscovitine derivatives BA12 or BP14 has also been shown to reduce tumor size to about one half in HCC models previously [24]. In good accordance, we find a reduction in the number of microvessels in a diethylnotrosamine (DEN)-induced orthotopic murine tumor model (Figure 1B), as well as in a Hep G2 xenograft murine tumor model (Figure 1C). Immunostaining for CD31 of a human HCC tissue microarray, in which we have previously demonstrated the overexpression of CDK5 [6] revealed a correlation of vascular density with CDK5 expression level in HCC patients (Figure 1D).

### HIF-1α and the transcription of its target genes is regulated by CDK5 in endothelial cells

**Figure 2:**
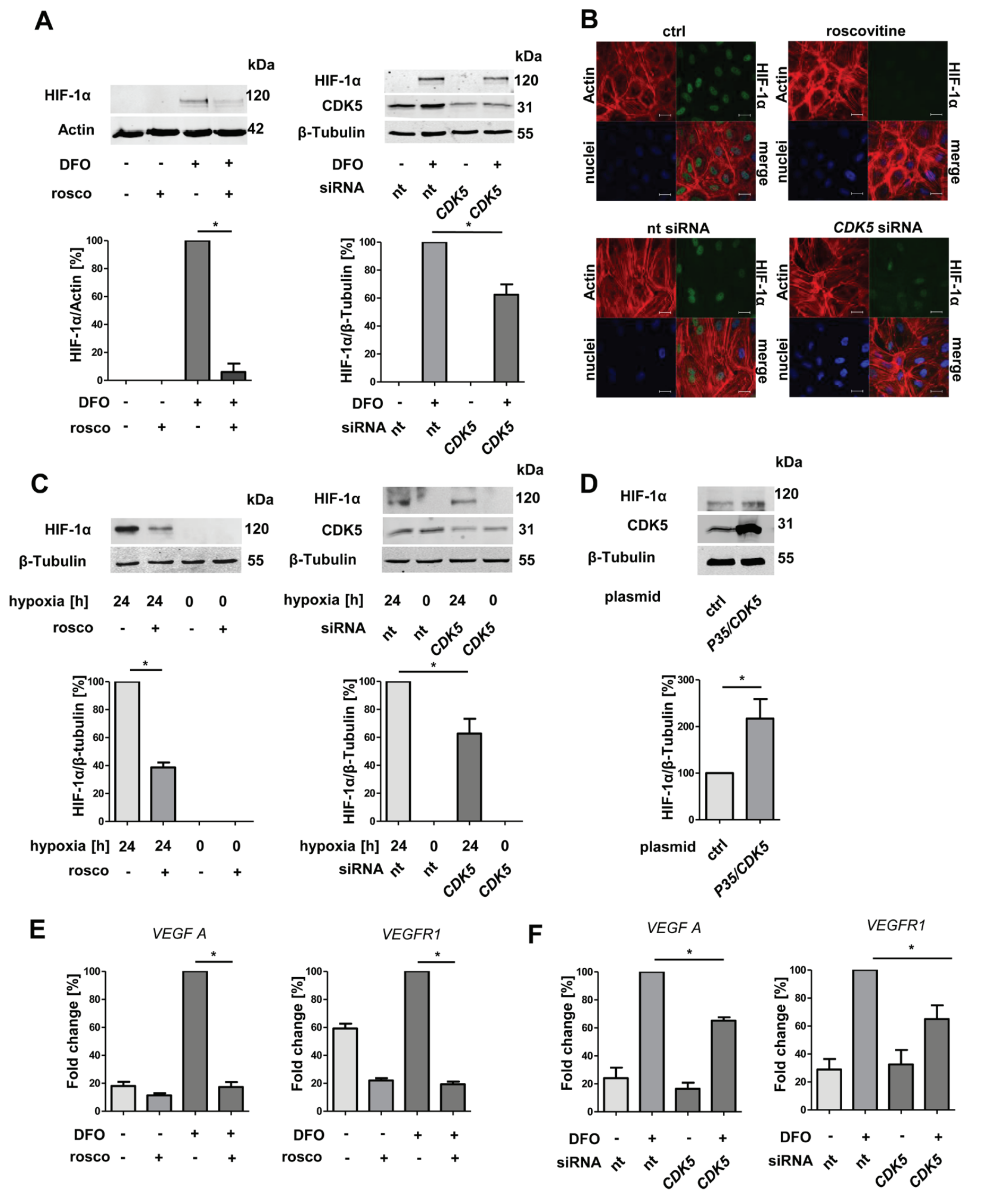
Protein level of HIF-1α and transcription of HIF-1α target genes correlate with CDK5 protein level in endothelial cells **A.** Immunoblots of HUVECs after treatment with DFO and subsequent either pharmacological inhibition (roscovitine 30 μM) or transient downregulation of CDK5 (upperpanels), and their densitometric quantifications (lower panels). One Way ANOVA on Newman-Keuls, *P < .05, *n* = 3. **B.** Immunostaining of HUVECs for HIF-1α, Actin and nuclei. Cells were either untreated or treated with 30 μM roscovitine or transfected with nt siRNA or *CDK5* siRNA. Scale bars: 20 μm. **C.** Upper panles: Immunoblots of lysates from HUVECs either incubated at 21% or 1% oxygen for 24 hours. CDK5 was inhibited by roscovitine or silenced with *CDK5* siRNA.Lower panels: Quantifications of the corresponding immunoblots are shown. One Way ANOVA on Newman-Keuls, *P < .05, *n* = 3. **D.** Immunoblot of HIF-1α in HUVECs either transfected with a control plasmid or with a *P35/CDK5* vector, cultivated under normoxic conditions. One Way ANOVA on Newman-Keuls, *P < .05, *n* = 3. **E.** Real-time PCR analysis of the HIF target genes *VEGFA* and *VEGFR1*. One Way ANOVA on Newman-Keuls, *P < .05, *n* = 3. **F.** Real-time PCR of *VEGFA* and *VEGFR1* for HUVECs either transfected with nt siRNA or *CDK5* siRNA, treated with 100 μM deferoxamine for six hours. One Way ANOVA on Newman-Keuls, * P < .05, *n* = 3. Error bars represent mean ± SEM. Nt stands for non targeting siRNA.

Hypoxia often promotes the expression of angiogenic factors in tumors by hypoxia inducible factors [25]. In addition, a potential link between HIF-1α and CDK5 has been postulated previously [26]. Therefore, we examined the effect of CDK5 inhibition or overexpression on HIF-1α. Hypoxia was induced in a hypoxic chamber (1% O2), or simulated by deferoxamine (DFO). Both, pharmacological inhibition of CDK5 by roscovitine or siRNA mediated downregulation, decreased the DFO- or hypoxia-induced level of HIF-1α in HUVECs (Figure 2A, 2B, 2C). Overexpression of P35/CDK5 increased HIF-1α levels under normoxic conditions (Figure 2D). To mimic the setting in hepatocellular carcinoma more closely, we further investigated hepatic sinusoidal endothelial cells, which are the source for angiogenesis in the liver. Pharmacological CDK5 inhibition as well as genetic down-regulation of CDK5 decreased HIF-1α protein level also in this cell type (Supplementary Figure 1).

To clarify whether CDK5 also influences the transcriptional activity of HIF-1α, we performed quantitative real-time RT-PCR analysis of angiogenesis relevant HIF-1α target genes *VEGFA* and *VEGFR1* in HUVECs. The hypoxia-induced increase of the *VEGFA* and *VEGFR1* mRNA level was reduced by pharmacological CDK5 inhibition (Figure 2E), as well as upon siRNA mediated down regulation (Figure 2F) of CDK5.

### Knockdown of CDK5 in the HCC cell line HUH7 inhibits angiogenesis *in vivo*

**Figure 3:**
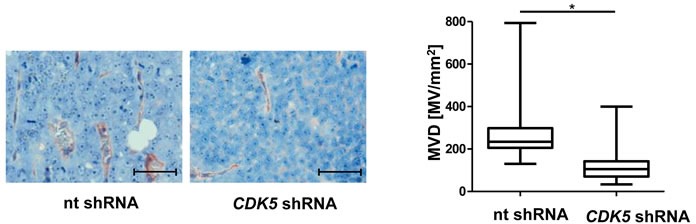
Knockdown of CDK5 in HUH7 cells reduces vascular density in HCC xenografts Immunostaining for CD31 (red) and haematoxylin (blue, nuclei) of tumors from non targeting (nt) shRNA HUH7 and *CDK5* shRNA HUH7 cells grown in SCID mice. MVD was determined by quantifying the number of microvessels in representative sections of the tumors. Scale bars: 50 μm. Non-parametric t-test on Mann-Whitney, *P < .05, *n* = 11. Whisker lines indicate maximum and minimum values.

We have recently shown that stable knockdown of CDK5 in HUH7 cells by shRNA reduces tumor growth in vivo [6]. Here we demonstrate the decreased vascularization of these xenografts with stable CDK5 shRNA HUH7 cells (Figure 3), and thus show that the anti-angiogenic effects of CDK5 reduction are not restricted to the endothelial cells, but do also originate in the HCC cells themselves.

### HIF-1α protein level and transcriptional activity are regulated by CDK5 in hepatocellular carcinoma cells

**Figure 4:**
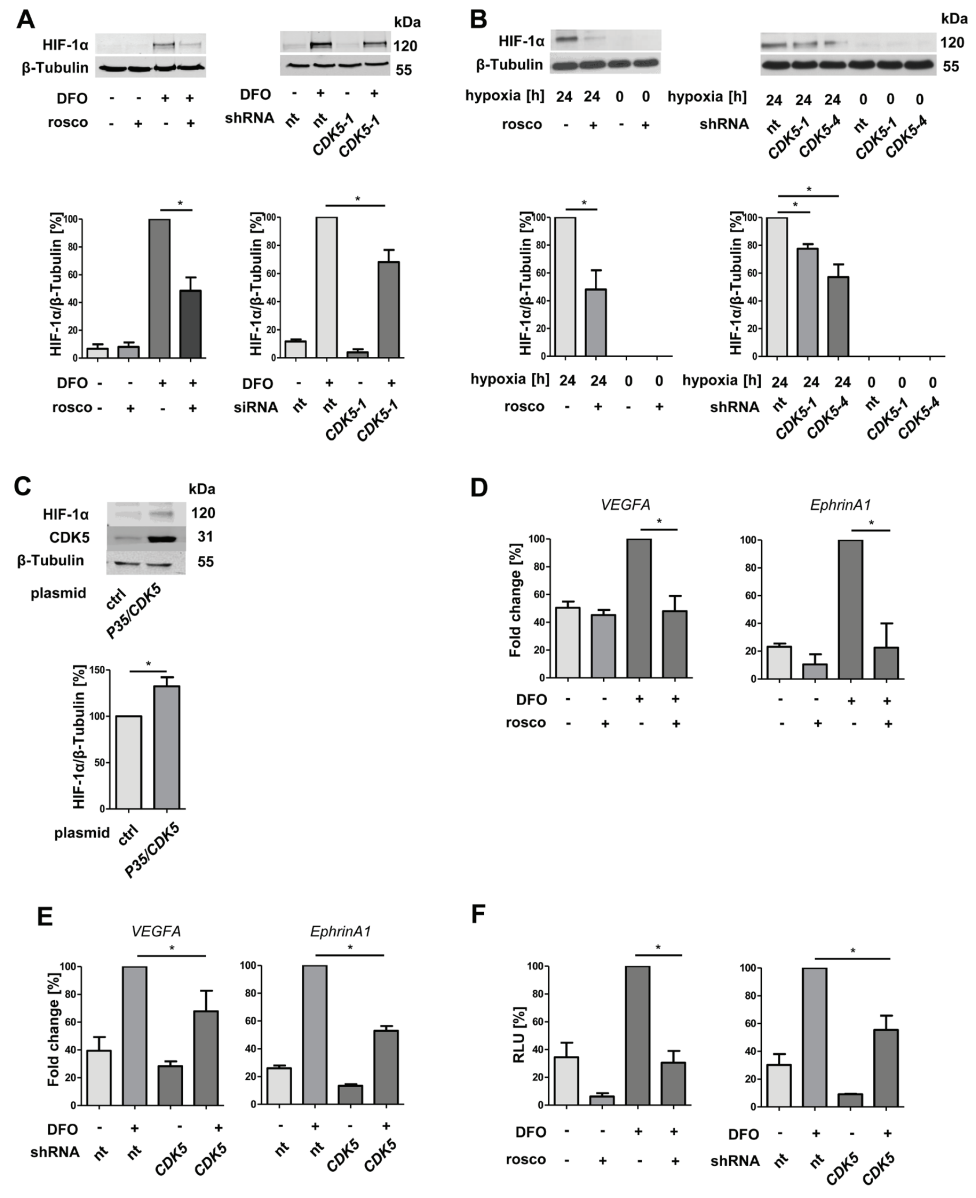
Protein level and transcriptional activity of HIF-1α correlate with CDK5 protein level in liver tumor cells **A.** Immunoblots show the protein levels of HIF-1α and β-Tubulin in HUH7 cells. CDK5 was inhibited by roscovitine (30 μM) or stably downregulated by *CDK5* shRNA. The ratio of HIF-1α to β-Tubulin is shown. One Way ANOVA on Newman-Keuls, *P < .05, *n* = 3. **B.** Immunoblots of either untreated or roscovitine pretreated HUH7 cells (30 μM, 30 minutes) or nt shRNA and *CDK5* shRNA HUH7 cells (CDK5-1 and CDK5-4: two independent clones 1 and 4) under hypoxia. One Way ANOVA on Newman-Keuls, *P < .05, *n* = 3. **C.** Protein level of HIF-1α, CDK5 and β-Tubulin in CDK5 overexpressing HUH7 cells under normoxia. One Way ANOVA on Newman-Keuls, *P < .05, *n* = 3. **D.** Real-time PCR of *VEGFA* and *EphrinA1*. HUH7 cells were either left untreated or treated with 30 μM roscovitine 30 minutes prior to deferoxamine (100 μM, 6 h). Graphs display the fold change in percent. One Way ANOVA on Newman-Keuls, *P < .05, *n* = 3. **E.** Real-time PCR of *VEGFA* and *EphrinA1* of nt shRNA HUH7 and *CDK5* shRNA HUH7 cells treated with 100 μM deferoxamine (6 h). One Way ANOVA on Newman-Keuls, *P < .05, *n* = 3. **F.** Dual luciferase reporter gene assay of HUH7 cells, transfected with a firefly luciferase vector *pGL4.27(HIF-REluc2P)* and a renilla luciferase vector *pGL4.74(hRluc/TK)*. CDK5 was either inhibited with roscovitine or by knockdown. Graphs show the relative light units (RLU) of firefly/renilla in percent. One Way ANOVA on Newman-Keuls, *P < .05, *n* = 3.

Since we have shown that a reduction of CDK5 in the HCC cells also inhibits angiogenesis, we extended our mechanistic work to HCC cells. CDK5 inhibition either by roscovitine or by stable knockdown (CDK5-1: CDK5 knockdown clone 1) led to a reduction of HIF-1α in DFO treated HUH7 cells (Figure 4A). This was be confirmed in two independent HUH7 clones, CDK5 knockdown clone 1 and clone 4 (CDK5-4, Supplementary Figure 2) cultivated under hypoxia (Figure 4B). Under normoxia P35/CDK5 overexpression led to an increase in HIF-1α level (Figure 4C), indicating a direct correlation between CDK5 and HIF-1α in HUH7 cells.

In good accordance, not only the protein level of the transcription factor is influenced by CDK5 inhibition, but also the transcription of its target genes *VEGFA* and *EphrinA1*. VEGFA and EphrinA1 are both important modulators of angiogenic processes [11, 27] and EphrinA1 has been shown to be highly upregulated in HCC, promoting cell growth of hepatocellular tumors [27]. Both transcripts were downregulated upon CDK5 inhibition (Figure 4D), as well as knockdown (Figure 4E). A HIF-1α reporter gene assay showed lower transcriptional activity of HIF-1α in roscovitine treated cells and in CDK5 knockdown clone 1 (Figure 4F).

### CDK5 inhibition reduces HIF-1α *in vivo*

**Figure 5:**
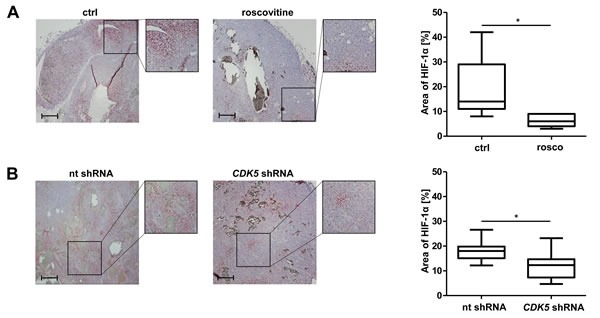
Pharmacological inhibition or distinct knockdown of CDK5 reduces the protein level of HIF-1α in a HUH7 xenograft tumor model **A.** Immunostaining of HIF-1α (red) and haematoxylin (blue, nuclei) of HCC tumors grown in SCID mice either treated with solvents or roscovitine. The graph shows the quantification of HIF-1α positive tumor areas of representative tissue sections. Scale bars: 200 μm. Non-parametric t-test on Mann-Whitney, *P < .05, *n* = 8. Whisker lines indicate maximum and minimum values. **B.** Immunostaining of HIF-1α (red) and haematoxylin (blue, nuclei) of tumors from nt shRNA HUH7 and *CDK5* shRNA HUH7 cells grown in SCID mice is shown. The graph shows the quantification of HIF-1α positive tumor areas of representative tissue sections. Scale bars: 200 μm. Non-parametric t-test on Mann-Whitney, *P < .05, *n* = 11. Whisker lines indicate maximum and minimum values.

In order to investigate the *in vivo* relevance of our proposed mode of action, we investigated HIF-1 α expression in HCC tissue by immunohistochemistry. Figure 5A shows a significant reduction in the area of HIF-1α positive cells for tumors of mice treated with roscovitine. Accordingly, we also observed this effect for tumors established from stable CDK5 shRNA HUH7 cells (Figure 5B).

### CDK5 interacts with HIF-1α, and prevents its proteasomal degradation

**Figure 6:**
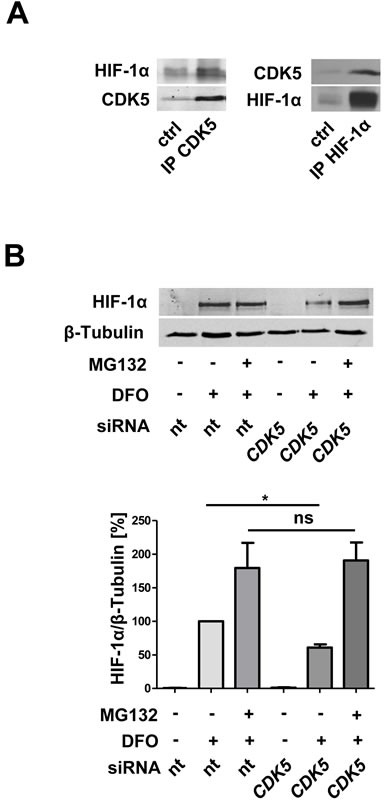
CDK5 directly interacts with HIF-1α and protects HIF-1α from proteasomal degradation **A.** Co-immunoprecipitation of CDK5 and HIF-1α in HUH7 cells, treated with 100 μM deferoxamine for six hours. *n* = 3 **B.** Immunoblot for HIF-1α and β-Tubulin of lysates from HUVECs, either transfected with nt siRNA or *CDK5* siRNA. Cells were left untreated or treated with 1 μM of the proteasomal inhibitor MG132, 30 minutes prior to deferoxamine (100 μM, six hours). The quantification summarizes the ratio of HIF-1α and β-Tubulin of three independent experiments. One Way ANOVA on Newman-Keuls, *P < .05, *n* = 3.

To investigate whether there is a direct interaction between CDK5 and HIF-1α co-immunoprecipitation experiments in both ways were performed, and, indeed revealed a protein-protein interaction (Figure 6A). Furthermore, inhibition of the proteasome completely rescues the effect of CDK5 inhibition on HIF-1α (Figure 6B), suggesting that CDK5 stabilizes HIF-1α via inhibition of proteasomal degradation.

### CDK5 phosphorylates HIF-1α at serine 687 causing its stabilization

**Figure 7:**
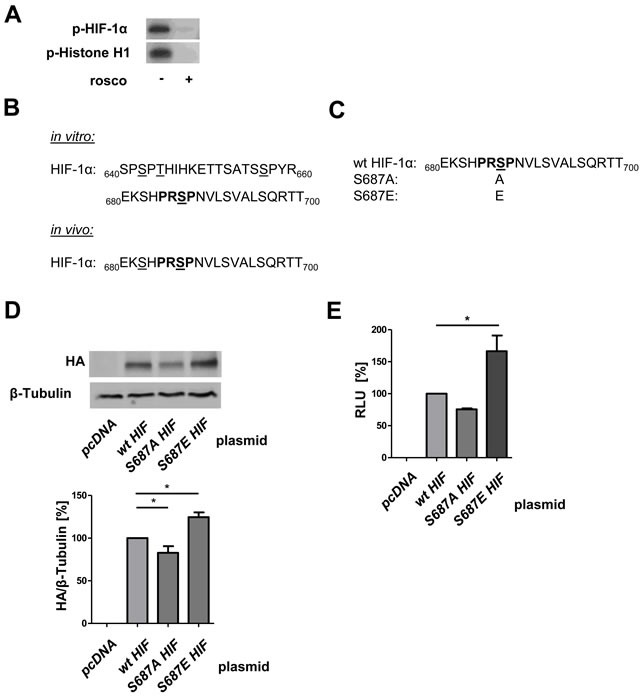
CDK5 phosphorylates HIF-1α at Serine 687 promoting its stability **A.** CDK5 kinase activity assay with recombinant P35/CDK5 and HIF-1α is shown, p-Histone H1 served as control. Samples were either left untreated or treated with 100 μM roscovitine. *n* = 3 **B.** Mass spectrometry analysis of CDK5 phosphorylation sites on HIF-1α is shown. CDK5 phosphorylation sites on either recombinant HIF-1α (*in vitro*) or immunoprecipitated HIF-1α *(in vivo*) from HUH7 cells, treated with DFO for 6 h, are displayed. Phosphorylated sites are underlined, CDK5 motif is indicated in bold. As negative control, non-phosphorylated HIF-1α was analysed. **C.** Peptide sequence of wildtype HIF-1α (wt) versus alanine (S687A) and glutamate (S687E) substituted HA-HIF-1α. Phosphorylation site of interest (serine 687) is underlined, CDK5 motif is indicated in bold. **D.** Immunoblot of HUH7 cells either transfected with a control plasmid (*pcDNA*), *wt HA-HIF-1α*, *S687A* or *S687E HA-HIF-1α* is shown. One Way ANOVA on Newman-Keuls, *P < .05, *n* = 3. **E.** Dual-Luciferase Assay of HUH7 cells either transfected with a control plasmid (*pcDNA*) or with *wt HA-HIF-1α*, *S687A* or *S687E HA-HIF-1α* is shown. Graph displays the RLU in per cent. One Way ANOVA on Newman-Keuls, *P < .05, *n* = 3.

In search of an underlying mechanism for the stabilization of HIF-1 α by CDK5, we performed point mutation studies. Recombinant P35/CDK5 phosphorylates recombinant HIF-1α *in vitro*, similar to the classical CDK5 substrate histone H1, and inhibitable by roscovitine (Figure 7A), showing HIF-1 α to be a direct substrate of CDK5. By mass spectrometry we identified serine 687 in recombinant HIF-1α, which lies within the CDK5 motif aa 685-688, to be phosphorylated by recombinant P35/CDK5. In an additional mass spectrometry approach, HIF-1α immunoprecipitated from DFO treated HUH7 cells also displayed a phosphorylation on serine 687 (Figure 7B, mass spectra see Supplementary Figures 3, 4). Point mutations of this site to either alanine (S687A) or glutamate (S687E) were generated in HA-HIF-1α with site-directed mutagenesis (Figure 7C, sequences see Supplementary Figure 5). In accordance with our other data S687A-HA-HIF-1α is less stable than wildtype (wt) HA-HIF-1α, whereas S687E-HA-HIF-1α shows an increased stability (Figure 7D). The reporter gene assay confirmed this result on a transcriptional level (Figure 7E).

### CDK5 is not activated by DFO, but is enriched in the nucleus

Treatment with DFO did not cause a direct increase of kinase activity of CDK5, as shown by kinase assay or quantification of the activator protein p35, as well as pY^419^c-src (Supplementary Figure 6A and 6B). However, quantitative analysis of subcellular CDK5 localization showed an enrichment of CDK5 in the nucleus (Supplementary Figure 6C).

## DISCUSSION

HCC is the third leading cause of cancer-related death in the world [28]. Since it is highly resistant to systemic therapies even after aggressive local therapy, there is an urgent need for new therapeutic options [29]. Hypervascularity is a characteristic feature of hepatocellular carcinoma and an increase in microvascular density is associated with a poor prognosis [30]. Angiogenesis is not only a fundamental step for tumor growth, but also for invasion and metastasis [31]. Therefore, in recent years, anti-angiogenic therapy has become of increasing importance in preclinical and clinical assessments [30]. Although there is evidence from preclinical studies that anti-angiogenic therapy inhibits the growth of HCC [32], many of these promising findings could not be confirmed in clinical trials [30]. Currently used anti-angiogenic drugs mainly target the VEGF pathway [11, 16]. However, the process of angiogenesis does not depend on a single molecule. Zeng *et al.* even suggest a VEGF/angiopoietin-independent tumor blood supply in HCC [33]. Additionally, there are several evasive mechanisms to anti-angiogenic VEGF therapy, like the activation of alternative pro-angiogenic pathways e.g. via hypoxia-inducible transcription factors [10, 26, 34]. Thus, novel approaches to anti-angiogenesis, less prone to development of resistances, are desperately needed. Along this line, we have recently suggested the serine/threonine kinase CDK5 as a promising target in endothelial cells: in addition to an anti-angiogenic effect of inhibition of CDK5 via the cytoskeletal effector Rac-1 [7], we found anti-inflammatory actions [8], and a role in lymphangiogenesis [9]. The latter two effects were caused by CDK5 induced alteration of transcription factors (NFkappaB and FOXC2, respectively). This, and the pronounced activity of CDK5 in the nucleus [6] make it tempting to speculate that also other transcriptional regulators might be controlled by CDK5, making it a unique signaling hub with such diverse downstream effects that development of resistances is very unlikely.

Hypoxia inducible factors are key players in cancer progression, enhancing proliferation, angiogenesis, metastasis, chemoresistance and radioresistance of HCC [35]. A correlation between the oxygen-dependent subunit HIF-1α and tumor size as well as a poor prognosis of HCC has already been shown [12]. Therefore, HIFs are interesting candidates for HCC treatment and some HIF inhibitors are in clinical trials or already approved [33]. There are some hints in the literature, which suggest a link between CDK5 and the transcription factor HIF-1α: First, Antoniou *at al.* have demonstrated an interaction between both proteins in mouse neuronal cells [38], however, the phosphorylation site they suggest due to a database research only exists in the murine but not in the human protein. Second, Xie *et al.* showed a correlation of CDK5 and VEGF expression in pituitary adenomas [36], however, without proposing an underlying mechanism, and third, several studies exist, which show an influence of multi-kinase inhibitors on HIF-1α [37], or HIF-dependent gene expression [38], again, without mechanistic explanation. In the present work we close this gap of knowledge by identifying Ser687 as the responsible phosphorylation site for Cdk5. Even though phosphorylation of HIF-1α can influence its transcriptional activity, subcellular localization, protein-protein interaction and stability [39], only few phosphorylation sites responsible for HIF-1α stabilization have been identified so far. Warfel *et al.* demonstrated that in colorectal cancer HIF-1α is phosphorylated at serine 668 by CDK1 enhancing the stability of the transcription factor [40]. Additionally, phosphorylation of HIF-1α at serine 696 by the serine/threonine kinase ataxia telangiectasia mutated (ATM) has been shown to stabilize HIF-1α down regulating mTOR-complex1 signaling in pediatric solid tumors [41]. These two phosphorylation sites lie within the inhibitory domain (aa 576-785) of HIF-1α, which inhibits its transactivation [42]. Interestingly, our newly identified phosphorylation site at serine 687 is located in close proximity to these sites.

Interestingly, the increased phosphorylation of HIF-1α after hypoxia seems not to be caused by an overall activation of CDK5, but by an enrichment of the kinase in the nucleus, where it co-localizes with HIF-1α.

In conclusion, we obtained compelling evidence that CDK5 directly stabilizes the transcription factor HIF-1α by phosphorylation, and thus promotes the formation of blood vessels. Together with our recent findings [6], which showed that CDK5 inhibition causes reduced HCC proliferation and clonogenic survival, this demonstrates the importance of CDK5 as an ideal and pharmacologically accessible therapeutic target with multiple modes of action in HCC.

## MATERIALS AND METHODS

### Compounds

(R)-roscovitine and deferoxamine were from Sigma-Aldrich, Taufkirchen, Germany. BA12 (2- [[[2- [(4-aminocyclohexyl)amino]-9-cyclopentyl-purin-6-yl]amino]methyl]-4-chloro-phenol) and BP14 (N2-(4-aminocyclohexyl)-9-cyclopentyl-N6- [[6-(2-furyl)-3-pyridyl]methyl]purine-2,6-diamine) were synthesized [43], dissolved and used [24] as previously described.

### Cell culture

HUH7 cells were obtained from the Japanese Collection of Research Bioresources (JCRB), and validated by the DSMZ (Deutsche Sammlung von Mikroorganismen und Zellkulturen) in Braunschweig, Germany. Cells were grown in Dulbecco's Modified Eagle Medium (DMEM, Sigma-Aldrich, Taufkirchen, Germany) containing 10% fetal calf serum (FCS, PAN Biotech, Aidenbach, Germany). HUVECs were purchased from PromoCell (Heidelberg, Germany), and grown in endothelial cell growth medium (ECGM, PromoCell, Heidelberg, Germany) supplemented with Penicillin (10.000 U/mL) and Streptomycin (10%).

### Immunohistochemistry

Immunostaining of tissue from murine tumor models: After explantation, tumors were cut in half, fixed in 4% paraformaldehyde, and embedded in paraffin. 5 μm thick sections from the center of the tumor were obtained and stained using the Vectastain® Universal Elite ABC Kit (Vector Laboratories, Burlingame, CA, USA) for antibody detection. AEC (Vector Laboratories, Burlingame, CA, USA) served as chromogen. Sections were incubated with primary antibodies (HIF-1α and CD31 1:100, BD Biosciences, Heidelberg, Germany) for 1 h at RT. Slides were counterstained with haematoxylin for 30 s. Mean vessel density was determined by counting the number of CD31 positive structures in 5 randomly chosen high power fields of 3 different slides per animal.

#### Immunostaining of a human tissue microarray (TMA)

Sections from a TMA of HCC patients, treated with liver transplantation or partial hepatectomy at the University Clinic Munich GroΔhadern between 1985 and 2008, were fluorescence stained. The tissue microarray was established as described previously [44]. Sections were incubated with a primary CD31 antibody (1:250, Abcam, Cambridge, UK) for 1 h at RT. Alexa Fluor 546 goat anti-rabbit IgG (H+L) (1:400, Invitrogen, Carlsbad, CA, USA) was used as secondary antibody (1 h, RT). Sections were additionally stained with Hoechst (5 μg/ml). CD31 staining was assessed using the following score: 0 – absent, 1 – weak, 2 – moderate, 3 – strong expression.

### Immunocytochemistry

HUVECs were fixed with 4% para-formaldehyde and permeabilized, followed by a blocking step. Slides were incubated with primary antibodies (CDK5 1:100, Invitrogen, Carlsbad, CA, USA; HIF-1α 1:100, BD Biosciences, Heidelberg, Germany) for 1 h at RT. Afterwards the secondary antibody goat anti-mouse Alexa Fluor 488 (1:400, Invitrogen, Carlsbad, CA, USA) was applied in combination with Hoechst (1:400, Sigma-Aldrich, Taufkirchen, Germany) and rhodamin-phalloidin (1:400, Invitrogen, Carlsbad, CA, USA) for 30 min at RT.

### Western blot

Western Blot was performed as previously described [45] (see Supplementary Materials).

### Quantitative real-time PCR

Real-time PCR was performed with 7300 Real Time PCR system (Applied Biosystems, Foster City, CA, USA). PCR components were supplied as master mix (Applied Biosystems, Foster City, CA, USA). For detecting the gene expression a set containing primer and probe for the specific gene was added (Applied Biosystems, Foster City, CA, USA). GAPDH served as housekeeping gene (biomers, Ulm, Germany).

### Kinase activity assay

Immunoprecipitated CDK5 or recombinant CDK5/P35 (20 ng, Millipore, Schwalbach, Germany) was diluted in 50 μl kinase buffer (50 mM HEPES pH 7.0, 10 mM MgCl_2_, 1 mM DTT, 1 mM NaF, 1 mM Na_3_VO_4_, 1 mM PMSF, 3 mM β-glycerophosphate, 4 mM Complete® EDTAfree). As substrates Histone H1 (2.5 μg, Sigma-Aldrich, Taufkirchen, Germany) or HIF-1α (2.5 μg, Abcam, Cambridge, UK) were added. 2 μM ATP and 10 μCi 32P-γ-ATP (Hartmann Analytic, Braunschweig, Germany) completed the reaction mix. Samples were incubated at 30°C for 20 minutes and prepared for SDS-Page gel electrophoresis. Phosphorylation of substrates was detected via autoradiography.

### Immunoprecipitation

Cells were lysed with a buffer containing 50 mM Tris/HCl pH 7.5, 250 mM NaCl, 1 mM EDTA pH 8.0, 10 mM NaF, 1x SIGMAFAST™ Protease Inhibitor. 2 μg antibody (Cdk5, Santa Cruz Biotechnology, Heidelberg, Germany) were added per 500 μg protein followed by an incubation overnight at 4°C. Each sample was incubated with 25 μL packed Protein G Agarose beads (Sigma-Aldrich, Taufkirchen, Germany) for 3 h at 4°C.

### Co-immunoprecipitation

Co-immunoprecipitation experiments were performed using the Pierce Crosslink Magnetic IP/Co-IP Kit (Thermo Scientific, Waltham, MA, USA) according to the manufacturer's protocol. 5 μg of CDK5 antibody (Invitrogen, Carlsbad, CA, USA) or HIF-1α antibody (BD Biosciences, Heidelberg, Germany) were used. Mouse IGg1 (Abcam, Cambridge, UK) served as control antibody.

### Mass spectrometry

LC-MS/MS was performed to detect phosphorylations on either recombinant HIF-1α (Abcam, Cambridge, UK) or HIF-1α immunoprecipitated in HUH7 cells (see supplementary documents).

### Site-directed mutagenesis

Point mutation in HIF-1α (Addgene, Cambridge, MA, USA, Kaelin W. [46]) at serine 687 was generated by site-directed mutagenesis with primers that contain specific mismatches: 5′GAACAGACAGAAAAATCTC ATCCAAGAGCTCCTAACGTGTTATCTGTCGCTTTG and 3′CAAAGCGACAGATAACACGTTAGGA GCTCTTGGATGAGATTTTTCTGTCTGTTC primers were used for alanine mutation (S687A), whereas 5′GAACAGACAGAAAAATCTCATCCAAG AGAGCCTAACGTGTTATCTGTCGCTTTG and 3′CAAAGCGACAGATAACACGTTAGGC TCTCTTGGATGAGATTTTTCTGTCTGTTC were used for glutamate mutation (S687E). The mutated codons are underlined.

### Dual-luciferase assay

HUH7 cells were seeded into a 96-well plate and transfected with pGL4.27(HIF-REluc2P) and pGL4.74(hRluc/TK) (Promega, Fitchburg, WI, USA). Activity of firefly and *Renilla* luciferases was determined using the Dual-Luciferase Reporter Assay System (Promega, Fitchburg, WI, USA) according to the manufacturer's instructions.

### Hypoxic chamber assay

Cells were cultivated in a hypoxic chamber (Don Whitley Scientific, Shipley, UK) at 1% O_2_ in carbonate-free medium (Biochrom AG, Berlin, Germany) supplemented with 10% FCS and Penicillin (10.000 U/mL) as well as Streptomycin (10%).

### *In vivo* experiments

The HUH7 xenograft tumor model was performed as described previously [6]. Briefly, either HUH7 cells or CDK5 knockdown HUH7 cells were subcutaneously injected into the flank of female SCID mice. Roscovitine treated mice were injected intraperitoneally (150 mg/kg) daily for seven days. Control mice received solvent (PBS/DMSO/Solutol 17:1:2) only.

The HG2 xenograft tumor model was performed as described previously [24]. HG2 cells were subcutaneously injected into SCID mice. Mice were injected intraperitoneally with either 5 mg/kg BA12 or 1 mg/kg BP14 daily for 17 days. Control mice received DMSO only.

The Diethylnitrosamine-induced orthotopic tumor model was performed as described previously [24]. For tumor development 14-day-old male C57BL/6J mice were intraperitoneally injected with a single dose of diethylnitrosamine (DEN, 25 mg/kg). After eight month of tumor growth, mice were injected intraperitoneally with either 5 mg/kg BA12 or 1 mg/kg BP14 in three cycles for 10 days. Control mice received DMSO only.

All *in vivo* experiments were performed according to Austrian guidelines or German legislation for the protection of animals and approved by the local government authorities.

### Statistics

Data are expressed as means ± standard error of mean (SEM) or as Whisker plots with lines indicating maximum and minimum values. The statistical significances were determined using GraphPad Prism 5. Statistical significance is assumed if *P ≤ .05*. Statistical tests are indicated in the corresponding figure legends.

## SUPPLEMENTARY MATERIAL FIGURES


