# Supplementary material for: Cyclin-dependent kinase 5 stabilizes hypoxia-inducible factor-1α: a novel approach for inhibiting angiogenesis in hepatocellular carcinoma
Source: Oncotarget. 2016 Mar 24;7(19):27108–21. doi: 10.18632/oncotarget.8342 (PMC5053636; doi:10.18632/oncotarget.8342)
Supplement: Supplementary file 1 [file oncotarget-07-27108-s001.pdf]

Cyclin-dependent kinase 5 stabilizes hypoxia-inducible factor-1α: a novel approach for inhibiting angiogenesis in hepatocellular carcinoma

Supplementary Material

Methods

Transfection procedures

HUVECs were either transfected with a Targefect/Virofect mixture used for siRNA or a Targefect/Peptide Enhancer mixture used for plasmids (Targeting Systems, USA) or by electroporation using Nucleofector™II (Amaxa, Germany) according to the manufacturer´s protocol. HUH7 cells were transfected with DharmaFECT Transfection reagent (Thermo Scientific, USA) following the manufacturer´s instructions.

CDK5 siRNA: CDK5 was silenced with an equal mixture of two different ON-TARGETplus CDK5 siRNAs (J-003239-09 and J-003239-10; Dharmacon, USA). ON-TARGETplus Non-targeting (nt) siRNA (D-001810-01; Dharmacon, USA) served as a control.

CDK5 shRNA: In HUH7 cells CDK5 silencing by shRNA was performed as previously described [1]. Cdk5 MISSION® shRNA Lentiviral Transduction Particles (Vector: pLKO.1-puro; SHCLNV-NM_004935; Clone ID: (1) TRCN0000021465, (2) TRCN0000021466, (3) TRCN0000021467, (4) TRCN0000194974, (5) TRCN0000195513; Sigma-Aldrich, Germany) and MISSION® pLKO.1-puro Non-Mammalian shRNA Control Transduction Particles (Sigma-Aldrich SHC002V, Germany) were used according to the manufacturer´s protocol.

CDK5 overexpression: HUVECs and HUH7 cells were cotransfected with 3 µg of CDK5-HA (Addgene 1872, van den Heuvel S. [2]) and P35 (Addgene 1347, Tsai Li-Huei), respectively. Transfection of 3 µg of pCMV-Neo-Bam (Addgene, 16440, Vogelstein B. [3]) served as a control.

S687A and S687E HA-HIF-1α: 3 µg of Alanine-mutated HIF (S687A) and glutamate-mutated HIF (S687E), generated by site-directed mutagenesis, were transfected into HUH7 cells followed by an incubation time of 24 h. Wildtype HIF-1α (wt, Addgene 18949, Kaelin W. [4]) and an empty pcDNA3 (Invitrogen, Germany) vector served as control (3 µg, 24 h). For Luciferase assay 0.3 µg of either pcDNA3, wt, S687A or S687E vector were used for transfection.

pGL4.27(HIF-REluc2P), pGL4.74(hRluc/TK): HUH7 cells were transfected with 3 µg of the firefly luciferase containing vector pGL4.27(HIF-REluc2P) (Promega, USA) and with 0.3 µg of the renilla luciferase containing vector pGL4.74(hRluc/TK) (Promega E692A, USA).

Western Blot

Membranes were incubated with primary antibodies overnight at 4°C (Actin, Santa Cruz sc-1615, 1:1000; β-Tubulin, Cell Signaling 2146, 1:1000; CDK5 Invitrogen AHZ0492, 1:1000; HA Covance MMS-101R, 1:1000; HIF-1α, BD Biosciences 610958, 1:750, Src , Cell Signaling 2110, 1:1000, phospho-Src (Tyr 416) Cell Signaling 6943, 1:1000). Dependent on the detection system the membranes were incubated with different secondary antibodies (2 h, RT): HRP-coupled (anti-mouse, Biozol, BZL07046; anti-rabbit, Dianova, 111-035-144) antibodies were used for chemiluminescence detection by x-ray films whereas IR-fluorescent Reagent conjugated antibodies (Invitrogen, A – 21057, A – 21109; LI-COR IRDye®, 926-32210D, 926-32211D) were used to detect the bands via fluorescence signal at the Odyssey Infrared Imaging system version 2.1 (LI-COR Biosciences, USA).

Mass spectrometry

Mass spectrometry of in vitro phosphorylated HIF-1α: 250 ng of HIF-1α (50 ng/µl) was reduced for 30 minutes with 0.5 µl 45 mM dithiothreitol and alkylated with 0.5 µl 100 mM iodoacetamide for 30 minutes in the dark. Tryptic digestion was done overnight with 5 ng trypsin (Promega,

Madison, USA) at 37°C. LC-MS/MS analysis was performed on a NanoLC Ultra system (Eksigent, CA, USA) coupled to an LTQ Orbitrap XL mass analyzer (Thermo Fisher Scientific, CA, USA). As mobile phase A 0.1% formic acid and as mobile phase B 84% acetonitrile/0.1% formic acid was used. The samples were injected onto a C18 trap column (C18 PepMap100, Particle size: 5 µm, 100 Å, Column size: 300 µm x 5 mm, Dionex, CA, USA) and separated on a reversed phase column (Reprosil-Pur C18 AQ, 3 µm; 150 mm x 75 µm, Dr. Maisch, Germany) at flow rate of 280 nl/min using two consecutive gradients (1 to 30% B in 120 minutes and 30 to 60% B in 10 minutes). Mass spectra were acquired in cycles of one MS Orbitrap scan followed by five data dependent ion trap MS/MS scans (CID, collision energy of 35%). MS data were analyzed with MASCOT 2.4 (Matrix Science, UK) using the human subset of the SwissProt Database and the following parameters: a) "Fixed modifications": Carbamidomethyl (C) b) Variable modifications: Oxidation (M); c) Decoy database: checked, d) Peptide charge: 2+ and 3+; e) Peptide tol. ±: 10 ppm; f) MS/MS tol. ±: 0.8 Da.

Mass spectrometry of immunoprecipitated HIF-1α: The gel slice containing HIF-1α was equilibrated twice with 50 mM NH_4HCO_3 for 10 minutes. For reduction and alkylation the gel slice was incubated in 45 mM dithiothreitol (30 minutes, 55 °C), followed by an incubation in 100 mM iodoacetamide (30 minutes). Tryptic digestion was performed overnight at 37°C with 100 ng porcine trypsin (Promega, WI, USA). Peptides were further extracted from the gel slices with 50 mM NH_4HCO_3 followed by a 80% acetonitrile wash. Chromatography was performed with an Ettan MDLC system (GE Healthcare, Germany) coupled to an LTQ ion trap mass spectrometer (Thermo Fisher Scientific, CA, USA). Injection was performed as described for the *in vitro* phosphorylated HIF-1α and separation on a Reprosil-Pur C18 AQ column (3 µm; 150 mm x 75 µm, Dr. Maisch, Germany) applying consecutive gradients from 1 to 30% B in 80 minutes and 30 to 60% B in 30 minutes at a flow rate of 280 nl/min (solvent A: 0.1% formic acid in water, solvent B: 0.1% formic acid in 84% acetonitrile). MS acquisition was performed in cycles of one MS-scan followed by three data dependent ion trap MS/MS scans (collision energy 35%). MS

data were analyzed as described for the *in vitro* phosphorylated HIF-1α sample with the difference that a peptide tolerance of ±: 2 Da was used.

Image Analysis

Fluorescence signals were quantified using ImageJ and integrated density of fluorescence detected in the nucleus compared to total fluorescence. For the analysis 8-bit pictures of CDK5 and Hoechst channels were used. Integrated density was first measured of the CDK5 channel representing the total fluorescence intensity. Hoechst Channel was then adjusted by applying Auto Threshold (Method Triangle) and colours inverted. By Image Calculator Hoechst Channel was substracted from CDK5 channel resulting in a new channel showing only the fluorescence signal in the nuclei of the cells. Integrated density was measured again and percentages of fluorescence intensity in the nuclei compared to total fluorescence calculated.

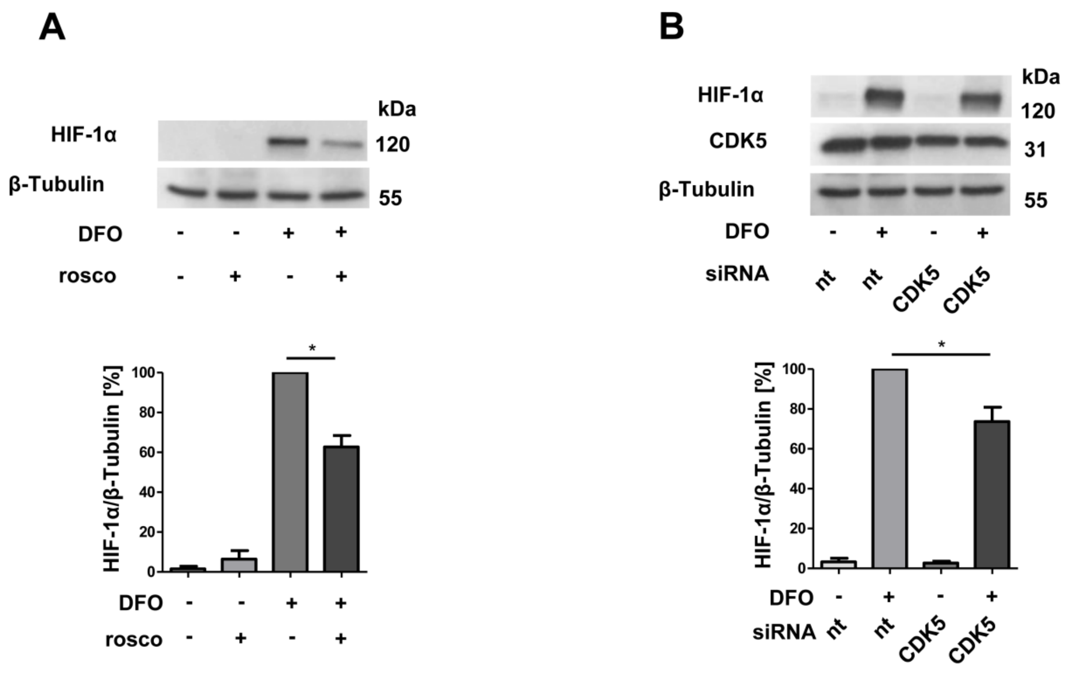

Supplementary Fig. 1: The protein level of HIF-1α also correlates with CDK5 protein level in human hepatic sinusoidal endothelial cells. (A) Immunoblots of lysates from HHSECs for HIF-1α and β-Tubulin. Cells were pretreated with roscovitine (30 µM) for 30 minutes, followed by DFO stimulation (100 µM) for 6 h. The quantification of the corresponding immunoblot is shown. One Way ANOVA on Newman-Keuls, * $P < .05$, n = 4. **(B)** Immunoblots of lysates from HHSECs for HIF-1α and β-Tubulin. CDK5 was transiently down-regulated by siRNA. Cells were stimulated with DFO (100 µM) for 6 h. The quantification of the corresponding immunoblot is shown. One Way ANOVA on Newman-Keuls, * $P < .05$, n = 4.

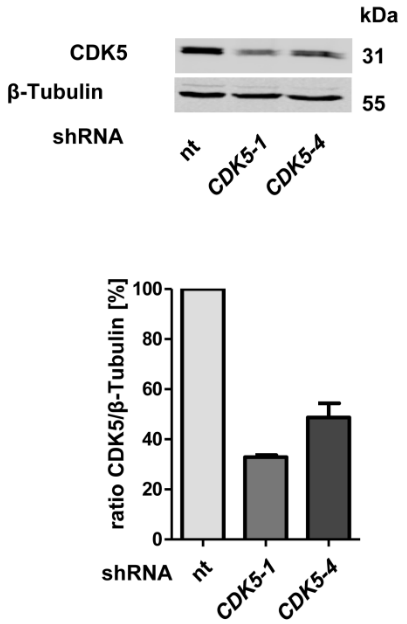

Supplementary Fig. 2: Knockdown level of two independent HUH7 CDK5 knockdown clones. Immunoblot for CDK5 and HIF-1α of lysates from HUH7 cells, either transfected with nt shRNA or *CDK5* shRNA is shown. The CDK5 knockdown level of CDK5 knockdown clone 1 and 4 in comparison to the control is summarized in the graph.

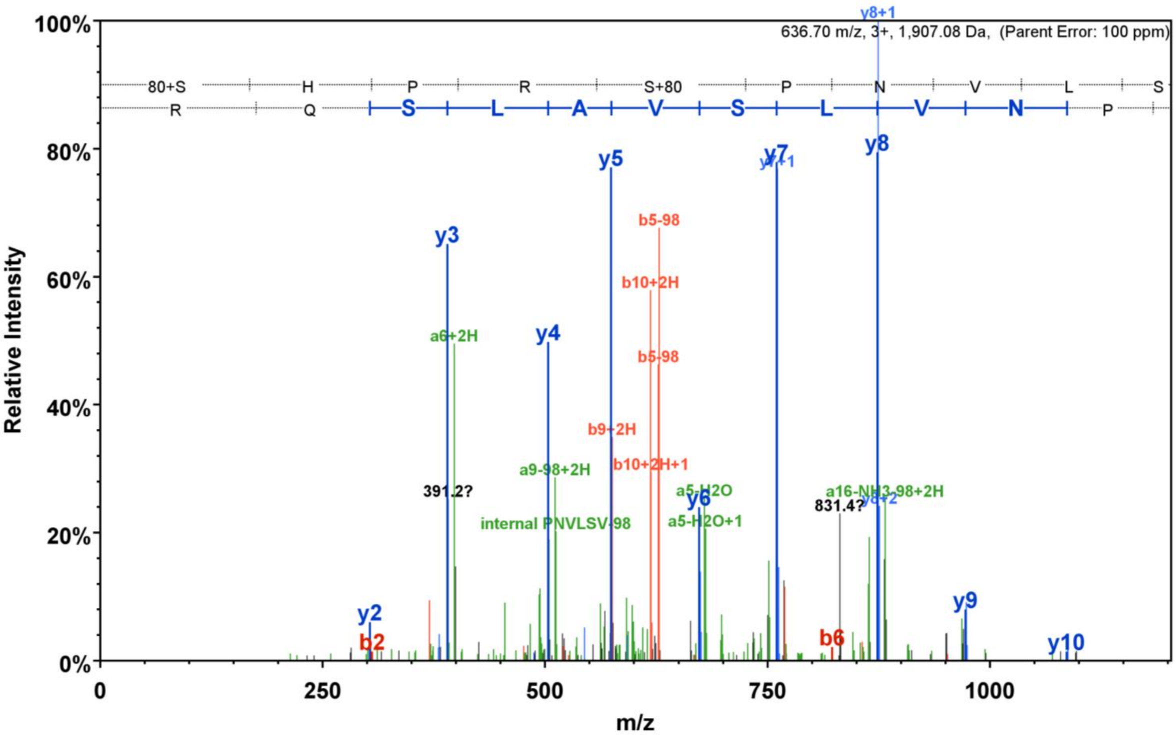

B	B Ions	B+2H	B-NH3	B-H2O	AA	Y Ions	Y+2H	Y-NH3	Y-H2O	Y
1	168.0	84.5		150.0	S+80	1,907.9	954.4	1,890.9	1,889.9	16
2	305.1	153.0		287.1	H	1,740.9	870.9	1,723.9	1,722.9	15
3	402.1	201.6		384.1	P	1,603.8	802.4	1,586.8	1,585.8	14
4	558.2	279.6	541.2	540.2	R	1,506.8	753.9	1,489.8	1,488.8	13
5	725.2	363.1	708.2	707.2	S+80	1,350.7	675.8	1,333.7	1,332.7	12
6	822.3	411.6	805.2	804.3	P	1,183.7	592.3	1,166.7	1,165.7	11
7	936.3	468.7	919.3	918.3	N	1,086.6	543.8	1,069.6	1,068.6	10
8	1,035.4	518.2	1,018.4	1,017.4	V	972.6	486.8	955.6	954.6	9
9	1,148.5	574.7	1,131.4	1,130.5	L	873.5	437.3	856.5	855.5	8
10	1,235.5	618.3	1,218.5	1,217.5	S	760.4	380.7	743.4	742.4	7
11	1,334.6	667.8	1,317.5	1,316.6	V	673.4	337.2	656.4	655.4	6
12	1,405.6	703.3	1,388.6	1,387.6	A	574.3	287.7	557.3	556.3	5
13	1,518.7	759.8	1,501.7	1,500.7	L	503.3	252.2	486.3	485.3	4
14	1,605.7	803.4	1,588.7	1,587.7	S	390.2	195.6	373.2	372.2	3
15	1,733.8	867.4	1,716.8	1,715.8	Q	303.2	152.1	286.2		2
16	1,907.9	954.4	1,890.9	1,889.9	R	175.1	88.1	158.1		1

Supplementary Fig. 3: MS/MS spectrum and fragmentation table of peptide $_{683}$**<u>S</u>**HPR**<u>S</u>**PNVLSVALSQR$_{698}$ **of HIF-1α.** The phosphorylated serines within the peptide sequence are underlined and marked as bold. Detected b ions are highlighted in red and y ions are highlighted in blue. Further signals which could be assigned to other fragment types are highlighted in green.

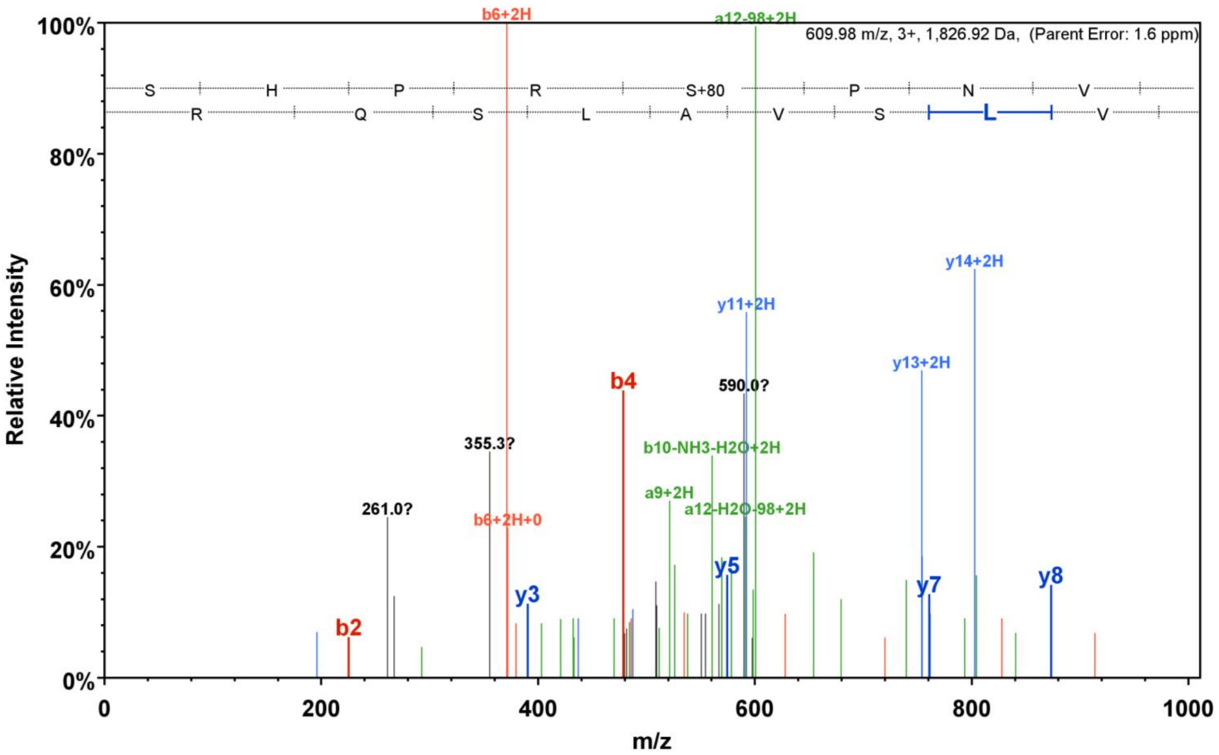

B	B Ions	B+2H	B-NH3	B-H2O	AA	Y Ions	Y+2H	Y-NH3	Y-H2O	Y
1	88.0	44.5		70.0	S	1,827.9	914.5	1,810.9	1,809.9	16
2	225.1	113.1		207.1	H	1,740.9	870.9	1,723.9	1,722.9	15
3	322.2	161.6		304.1	P	1,603.8	802.4	1,586.8	1,585.8	14
4	478.3	239.6	461.2	460.2	R	1,506.8	753.9	1,489.8	1,488.8	13
5	645.3	323.1	628.2	627.2	S+80	1,350.7	675.8	1,333.7	1,332.7	12
6	742.3	371.7	725.3	724.3	P	1,183.7	592.3	1,166.7	1,165.7	11
7	856.3	428.7	839.3	838.3	N	1,086.6	543.8	1,069.6	1,068.6	10
8	955.4	478.2	938.4	937.4	V	972.6	486.8	955.6	954.6	9
9	1,068.5	534.8	1,051.5	1,050.5	L	873.5	437.3	856.5	855.5	8
10	1,155.5	578.3	1,138.5	1,137.5	S	760.4	380.7	743.4	742.4	7
11	1,254.6	627.8	1,237.6	1,236.6	V	673.4	337.2	656.4	655.4	6
12	1,325.6	663.3	1,308.6	1,307.6	A	574.3	287.7	557.3	556.3	5
13	1,438.7	719.9	1,421.7	1,420.7	L	503.3	252.2	486.3	485.3	4
14	1,525.8	763.4	1,508.7	1,507.7	S	390.2	195.6	373.2	372.2	3
15	1,653.8	827.4	1,636.8	1,635.8	Q	303.2	152.1	286.2		2
16	1,827.9	914.5	1,810.9	1,809.9	R	175.1	88.1	158.1		1

Supplementary Fig.4: MS/MS spectrum and fragmentation table of peptide $_{683}$SHPR**<u>S</u>**PNVLSVALSQR$_{698}$ **of HIF-1α.** The phosphorylated serine within the peptide sequence is underlined and marked as bold. Detected b ions are highlighted in red and y ions are highlighted in blue. Further signals which could be assigned to other types of fragments are highlighted in green.

S687A:

```
CLUSTAL 2.1 multiple sequence alignment


WT              TCCGCCCCATTGACGCAAATGGGCGGTAGGCGTGTACGGTGGGAGGTCTATATAAGCAGA 60
XL1A1           -----------------------------------------------------AAGCAGA 7
                                                                     *******

WT              GCTCTCTGGCTAACTAGAGAACCCACTGCTTACTGGCTTATCGAAATTAATACGACTCAC 120
XL1A1           GCTCTCTGGCTAACTAGAGAACCCACTGCTTACTGGCTTATCGAAATTAATACGACTCAC 67
                ************************************************************

WT              TATAGGGAGACCCAAGCTTACCATGGCCTACCCNTACGACGTGCCCGACTACGCCTCCCT 180
XL1A1           TATAGGGAGACCCAAGCTTACCATGGCCTACCCCTACGACGTGCCCGACTACGCCTCCCT 127
                ********************************* **************************

WT              CGGATCCGCCACCATGGAGGGCGCCGGCGGCGCGAACGACAAGAAAAAGATAAGTTCTGA 240
XL1A1           CGGATCCGCCACCATGGAGGGCGCCGGCGGCGCGAACGACAAGAAAAAGATAAGTTCTGA 187
                ************************************************************

WT              ACGTCGAAAAGAAAAGTCTCGAGATGCAGCCAGATCTCGGCGAAGTAAAGAATCTGAAGT 300
XL1A1           ACGTCGAAAAGAAAAGTCTCGAGATGCAGCCAGATCTCGGCGAAGTAAAGAATCTGAAGT 247
                ************************************************************

WT              TTTTTATGAGCTTGCTCATCAGTTGCCACTTCCACATAATGTGAGTTCGCATCTTGATAA 360
XL1A1           TTTTTATGAGCTTGCTCATCAGTTGCCACTTCCACATAATGTGAGTTCGCATCTTGATAA 307
                ************************************************************

WT              GGCCTCTGTGATGAGGCTTACCATCAGCTATTTGCGTGTGAGGAAACTTCTGGATGCTGG 420
XL1A1           GGCCTCTGTGATGAGGCTTACCATCAGCTATTTGCGTGTGAGGAAACTTCTGGATGCTGG 367
                ************************************************************

WT              TGATTTGGATATTGAAGATGACATGAAAGCACAGATGAATTGCTTTTATTTGAAAGCCTT 480
XL1A1           TGATTTGGATATTGAAGATGACATGAAAGCACAGATGAATTGCTTTTATTTGAAAGCCTT 427
                ************************************************************

WT              GGATGGTTTTGTTATGGTTCTCACAGATGATGGTGACATGATTTACATTTCTGATAATGT 540
XL1A1           GGATGGTTTTGTTATGGTTCTCACAGATGATGGTGACATGATTTACATTTCTGATAATGT 487
                ************************************************************

WT              GAACAAATACATGGGATTAACTCAGTTTGAACTAACTGGACACAGTGTGTTTGATTTTAC 600
XL1A1           GAACAAATACATGGGATTAACTCAGTTTGAACTAACTGGACACAGTGTGTTTGATTTTAC 547
                ************************************************************

WT              TCATCCATGTGACCATGAGGAAATGAGAGAAATGCTTACACACAGAAATGGCCTTGTGAA 660
XL1A1           TCATCCATGTGACCATGAGGAAATGAGAGAAATGCTTACACACAGAAATGGCCTTGTGAA 607
                ************************************************************

WT              AAAGGGTAAAGAACAAAACACACAGCGAAGCTTTTTTCTCAGAATGAAGTGTACCCTAAC 720
XL1A1           AAAGGGTAAAGAACAAAACACACAGCGAAGCTTTTTTCTCAGAATGAAGTGTACCCTAAC 667
                ************************************************************

WT              TAGCCGAGGAAGAACTATGAACATAAAGTCTGCAACATGGAAGGTATTGCACTGCACAGG 780
XL1A1           TAGCCGAGGAAGAACTATGAACATAAAGTCTGCAACATGGAAGGTATTGCACTGCACAGG 727
                ************************************************************

WT              CCACATTCACGTATATGATACCAACAGTAACCAACCTCAGTGTGGGTATAAGAAACCACC 840
XL1A1           CCACATTCACGTATATGATACCAACAGTAACCAACCTCAGTGTGGGTATAAGAAACCACC 787
                ************************************************************

WT              TATGACCTGCTTGGTGCTGATTTGTGAACCCATTCCTCACCCATCAAATATTGAAATTCC 900
XL1A1           TATGACCTGCTTGGTGCTGATTTGTGAACCCATTCCTCACCCATCAAATATTGAAATTCC 847
                ************************************************************
```

```
WT              TTTAGATAGCAAGACTTTCCTCAGTCGACACAGCCTGGATATGAAATTTTCTTATTGTGA 960
XL1A1           TTTAGATAGCAAGACTTTCCTCAGTCGACACAGCCTGGATATGAAATTTTCTTATTGTGA 907
                ************************************************************

WT              TGAAAGAATTACCGAATTGATGGGATATGAGCCAGAAGAACTTTTAGGCCGCTCAATTTA 1020
XL1A1           TGAAAGAATTACCGAATTGATGGGATATGAGCCAGAAGAACTTTTAGGCCGCTCAATTTA 967
                ************************************************************

WT              TGAATATTATCATGCTTTGGACTCTGATCATCTGACCAAAACTCATCATGATATGTTTAC 1080
XL1A1           TGAATATTATCATGCTTTGGACTCTGATCATCTGACCAAAACTCATCATGATATGTTTAC 1027
                ************************************************************

WT              TAAAGGACAAGTCACCACAGGACAGTACAGGATGCTTGCCAAAAGAGGTGGATATGTCTG 1140
XL1A1           TAAAGGACAAGTCACCACAGGACAGTACAGGATGCTTGCCAAAAGAGGTGGATATGTCTG 1087
                ************************************************************

WT              GGTTGAAACTCAAGCAACTGTCATATATAACACCAAGAATTCTCAACCACAGTGCATTGT 1200
XL1A1           GGTTGAAACTCAAGCAACTGTCATATATAACACCAAGAATTCTCAACCACAGTGCATTGT 1147
                ************************************************************

WT              ATGTGTGAATTACGTTGTGAGTGGTATTATTCAGCACGACTTGATTTTCTCCCTTCAACA 1260
XL1A1           ATGTGTGAATTACGTTGTGAGTGGTATTATTCAGCACGACTTGATTTTCTCCCTTCAACA 1207
                ************************************************************

WT              AACAGAATGTGTCCTTAAACCGGTTGAATCTTCAGATATGAAAATGACTCAGCTATTCAC 1320
XL1A1           AACAGAATGTGTCCTTAAACCGGTTGAATCTTCAGATATGAAAATGACTCAGCTATTCAC 1267
                ************************************************************

WT              CAAAGTTGAATCAGAAGATACAAGTAGCCTCTTTGACAAACTTAAGAAGGAACCTGATGC 1380
XL1A1           CAAAGTTGAATCAGAAGATACAAGTAGCCTCTTTGACAAACTTAAGAAGGAACCTGATGC 1327
                ************************************************************

WT              TTTAACTTTGCTGGCCCCAGCCGCTGGAGACACAATCATATCTTTAGATTTTGGCAGCAA 1440
XL1A1           TTTAACTTTGCTGGCCCCAGCCGCTGGAGACACAATCATATCTTTAGATTTTGGCAGCAA 1387
                ************************************************************

WT              CGACACAGAAACTGATGACCAGCAACTTGAGGAAGTACCATTATATAATGATGTAATGCT 1500
XL1A1           CGACACAGAAACTGATGACCAGCAACTTGAGGAAGTACCATTATATAATGATGTAATGCT 1447
                ************************************************************

WT              CCCCTCACCCAACGAAAAATTACAGAATATAAATTTGGCAATGTCTCCATTACCCACCGC 1560
XL1A1           CCCCTCACCCAACGAAAAATTACAGAATATAAATTTGGCAATGTCTCCATTACCCACCGC 1507
                ************************************************************

WT              TGAAACGCCAAAGCCACTTCGAAGTAGTGCTGACCCTGCACTCAATCAAGAAGTTGCATT 1620
XL1A1           TGAAACGCCAAAGCCACTTCGAAGTAGTGCTGACCCTGCACTCAATCAAGAAGTTGCATT 1567
                ************************************************************

WT              AAAATTAGAACCAAATCCAGAGTCACTGGAACTTTCTTTTACCATGCCCCAGATTCAGGA 1680
XL1A1           AAAATTAGAACCAAATCCAGAGTCACTGGAACTTTCTTTTACCATGCCCCAGATTCAGGA 1627
                ************************************************************

WT              TCAGACACCTAGTCCTTCCGATGGAAGCACTAGACAAAGTTCACCTGAGCCTAATAGTCC 1740
XL1A1           TCAGACACCTAGTCCTTCCGATGGAAGCACTAGACAAAGTTCACCTGAGCCTAATAGTCC 1687
                ************************************************************

WT              CAGTGAATATTGTTTTTATGTGGATAGTGATATGGTCAATGAATTCAAGTTGGAATTGGT 1800
XL1A1           CAGTGAATATTGTTTTTATGTGGATAGTGATATGGTCAATGAATTCAAGTTGGAATTGGT 1747
                ************************************************************

WT              AGAAAAACTTTTTGCTGAAGACACAGAAGCAAAGAACCCATTTTCTACTCAGGACACAGA 1860
XL1A1           AGAAAAACTTTTTGCTGAAGACACAGAAGCAAAGAACCCATTTTCTACTCAGGACACAGA 1807
                ************************************************************
```

```
WT              TTTAGACTTGGAGATGTTAGCTCCCTATATCCCAATGGATGATGACTTCCAGTTACGTTC 1920
XL1A1           TTTAGACTTGGAGATGTTAGCTCCCTATATCCCAATGGATGATGACTTCCAGTTACGTTC 1867
                ************************************************************

WT              CTTCGATCAGTTGTCACCATTAGAAAGCAGTTCCGCAAGCCCTGAAAGCGCAAGTCCTCA 1980
XL1A1           CTTCGATCAGTTGTCACCATTAGAAAGCAGTTCCGCAAGCCCTGAAAGCGCAAGTCCTCA 1927
                ************************************************************

WT              AAGCACAGTTACAGTATTCCAGCAGACTCAAATACAAGAACCTACTGCTAATGCCACCAC 2040
XL1A1           AAGCACAGTTACAGTATTCCAGCAGACTCAAATACAAGAACCTACTGCTAATGCCACCAC 1987
                ************************************************************

WT              TACCACTGCCACCACTGATGAATTAAAAACAGTGACAAAAGACCGTATGGAAGACATTAA 2100
XL1A1           TACCACTGCCACCACTGATGAATTAAAAACAGTGACAAAAGACCGTATGGAAGACATTAA 2047
                ************************************************************

WT              AATATTGATTGCATCTCCATCTCCTACCCACATACATAAAGAAACTACTAGTGCCACATC 2160
XL1A1           AATATTGATTGCATCTCCATCTCCTACCCACATACATAAAGAAACTACTAGTGCCACATC 2107
                ************************************************************

WT              ATCACCATATAGAGATACTCAAAGTCGGACAGCCTCACCAAACAGAGCAGGAAAAGGAGT 2220
XL1A1           ATCACCATATAGAGATACTCAAAGTCGGACAGCCTCACCAAACAGAGCAGGAAAAGGAGT 2167
                ************************************************************

WT              CATAGAACAGACAGAAAAATCTCATCCAAGAAGCCCTAACGTGTTATCTGTCGCTTTGAG 2280
XL1A1           CATAGAACAGACAGAAAAATCTCATCCAAGAGCTCCTAACGTGTTATCTGTCGCTTTGAG 2227
                *******************************.  **************************

WT              TCAAAGAACTACAGTTCCTGAGGAAGAACTAAATCCAAAGATACTAGCTTTGCAGAATGC 2340
XL1A1           TCAAAGAACTACAGTTCCTGAGGAAGAACTAAATCCAAAGATACTAGCTTTGCAGAATGC 2287
                ************************************************************

WT              TCAGAGAAAGCGAAAAATGGAACATGATGGTTCACTTTTTCAAGCAGTAGGAATTGGAAC 2400
XL1A1           TCAGAGAAAGCGAAAAATGGAACATGATGGTTCACTTTTTCAAGCAGTAGGAATTGGAAC 2347
                ************************************************************

WT              ATTATTACAGCAGCCAGACGATCATGCAGCTACTACATCACTTTCTTGGAAACGTGTAAA 2460
XL1A1           ATTATTACAGCAGCCAGACGATCATGCAGCTACTACATCACTTTCTTGGAAACGTGTAAA 2407
                ************************************************************

WT              AGGATGCAAATCTAGTGAACAGAATGGAATGGAGCAAAAGACAATTATTTTAATACCCTC 2520
XL1A1           AGGATGCAAATCTAGTGAACAGAATGGAATGGAGCAAAAGACAATTATTTTAATACCCTC 2467
                ************************************************************

WT              TGATTTAGCATGTAGACTGCTGGGGCAATCAATGGATGAAAGTGGATTACCACAGCTGAC 2580
XL1A1           TGATTTAGCATGTAGACTGCTGGGGCAATCAATGGATGAAAGTGGATTACCACAGCTGAC 2527
                ************************************************************

WT              CAGTTATGATTGTGAAGTTAATGCTCCTATACAAGGCAGCAGAAACCTACTGCAGGGTGA 2640
XL1A1           CAGTTATGATTGTGAAGTTAATGCTCCTATACAAGGCAGCAGAAACCTACTGCAGGGTGA 2587
                ************************************************************

WT              AGAATTACTCAGAGCTTTGGATCAAGTTAACTGACAATTCTGCAGATATCCATCACACTG 2700
XL1A1           AGAATTACTCAGAGCTTTGGATCAAGTTAACTGACAATTCTGCAGATATCCATCACACTG 2647
                ************************************************************

WT              GCGGCCGCTCGAGCATGCATCTAGAGG 2727
XL1A1           GCGGCC--------------------- 2653
```

S687E:

```
CLUSTAL 2.1 multiple sequence alignment


WT              TCCGCCCCATTGACGCAAATGGGCGGTAGGCGTGTACGGTGGGAGGTCTATATAAGCAGA 60
XL1E11          --------------------------------------------------------CAGA 4
                                                                        ****

WT              GCTCTCTGGCTAACTAGAGAACCCACTGCTTACTGGCTTATCGAAATTAATACGACTCAC 120
XL1E11          GCTCTCTGGCTAACTAGAGAACCCACTGCTTACTGGCTTATCGAAATTAATACGACTCAC 64
                ************************************************************

WT              TATAGGGAGACCCAAGCTTACCATGGCCTACCCNTACGACGTGCCCGACTACGCCTCCCT 180
XL1E11          TATAGGGAGACCCAAGCTTACCATGGCCTACCCCTACGACGTGCCCGACTACGCCTCCCT 124
                ********************************* **************************

WT              CGGATCCGCCACCATGGAGGGCGCCGGCGGCGCGAACGACAAGAAAAAGATAAGTTCTGA 240
XL1E11          CGGATCCGCCACCATGGAGGGCGCCGGCGGCGCGAACGACAAGAAAAAGATAAGTTCTGA 184
                ************************************************************

WT              ACGTCGAAAAGAAAAGTCTCGAGATGCAGCCAGATCTCGGCGAAGTAAAGAATCTGAAGT 300
XL1E11          ACGTCGAAAAGAAAAGTCTCGAGATGCAGCCAGATCTCGGCGAAGTAAAGAATCTGAAGT 244
                ************************************************************

WT              TTTTTATGAGCTTGCTCATCAGTTGCCACTTCCACATAATGTGAGTTCGCATCTTGATAA 360
XL1E11          TTTTTATGAGCTTGCTCATCAGTTGCCACTTCCACATAATGTGAGTTCGCATCTTGATAA 304
                ************************************************************

WT              GGCCTCTGTGATGAGGCTTACCATCAGCTATTTGCGTGTGAGGAAACTTCTGGATGCTGG 420
XL1E11          GGCCTCTGTGATGAGGCTTACCATCAGCTATTTGCGTGTGAGGAAACTTCTGGATGCTGG 364
                ************************************************************

WT              TGATTTGGATATTGAAGATGACATGAAAGCACAGATGAATTGCTTTTATTTGAAAGCCTT 480
XL1E11          TGATTTGGATATTGAAGATGACATGAAAGCACAGATGAATTGCTTTTATTTGAAAGCCTT 424
                ************************************************************

WT              GGATGGTTTTGTTATGGTTCTCACAGATGATGGTGACATGATTTACATTTCTGATAATGT 540
XL1E11          GGATGGTTTTGTTATGGTTCTCACAGATGATGGTGACATGATTTACATTTCTGATAATGT 484
                ************************************************************

WT              GAACAAATACATGGGATTAACTCAGTTTGAACTAACTGGACACAGTGTGTTTGATTTTAC 600
XL1E11          GAACAAATACATGGGATTAACTCAGTTTGAACTAACTGGACACAGTGTGTTTGATTTTAC 544
                ************************************************************

WT              TCATCCATGTGACCATGAGGAAATGAGAGAAATGCTTACACACAGAAATGGCCTTGTGAA 660
XL1E11          TCATCCATGTGACCATGAGGAAATGAGAGAAATGCTTACACACAGAAATGGCCTTGTGAA 604
                ************************************************************

WT              AAAGGGTAAAGAACAAAACACACAGCGAAGCTTTTTTCTCAGAATGAAGTGTACCCTAAC 720
XL1E11          AAAGGGTAAAGAACAAAACACACAGCGAAGCTTTTTTCTCAGAATGAAGTGTACCCTAAC 664
                ************************************************************

WT              TAGCCGAGGAAGAACTATGAACATAAAGTCTGCAACATGGAAGGTATTGCACTGCACAGG 780
XL1E11          TAGCCGAGGAAGAACTATGAACATAAAGTCTGCAACATGGAAGGTATTGCACTGCACAGG 724
                ************************************************************

WT              CCACATTCACGTATATGATACCAACAGTAACCAACCTCAGTGTGGGTATAAGAAACCACC 840
XL1E11          CCACATTCACGTATATGATACCAACAGTAACCAACCTCAGTGTGGGTATAAGAAACCACC 784
                ************************************************************

WT              TATGACCTGCTTGGTGCTGATTTGTGAACCCATTCCTCACCCATCAAATATTGAAATTCC 900
XL1E11          TATGACCTGCTTGGTGCTGATTTGTGAACCCATTCCTCACCCATCAAATATTGAAATTCC 844
                ************************************************************
```

```
WT          TTTAGATAGCAAGACTTTCCTCAGTCGACACAGCCTGGATATGAAATTTTCTTATTGTGA 960
XL1E11      TTTAGATAGCAAGACTTTCCTCAGTCGACACAGCCTGGATATGAAATTTTCTTATTGTGA 904
            ************************************************************

WT          TGAAAGAATTACCGAATTGATGGGATATGAGCCAGAAGAACTTTTAGGCCGCTCAATTTA 1020
XL1E11      TGAAAGAATTACCGAATTGATGGGATATGAGCCAGAAGAACTTTTAGGCCGCTCAATTTA 964
            ************************************************************

WT          TGAATATTATCATGCTTTGGACTCTGATCATCTGACCAAAACTCATCATGATATGTTTAC 1080
XL1E11      TGAATATTATCATGCTTTGGACTCTGATCATCTGACCAAAACTCATCATGATATGTTTAC 1024
            ************************************************************

WT          TAAAGGACAAGTCACCACAGGACAGTACAGGATGCTTGCCAAAAGAGGTGGATATGTCTG 1140
XL1E11      TAAAGGACAAGTCACCACAGGACAGTACAGGATGCTTGCCAAAAGAGGTGGATATGTCTG 1084
            ************************************************************

WT          GGTTGAAACTCAAGCAACTGTCATATATAACACCAAGAATTCTCAACCACAGTGCATTGT 1200
XL1E11      GGTTGAAACTCAAGCAACTGTCATATATAACACCAAGAATTCTCAACCACAGTGCATTGT 1144
            ************************************************************

WT          ATGTGTGAATTACGTTGTGAGTGGTATTATTCAGCACGACTTGATTTTCTCCCTTCAACA 1260
XL1E11      ATGTGTGAATTACGTTGTGAGTGGTATTATTCAGCACGACTTGATTTTCTCCCTTCAACA 1204
            ************************************************************

WT          AACAGAATGTGTCCTTAAACCGGTTGAATCTTCAGATATGAAAATGACTCAGCTATTCAC 1320
XL1E11      AACAGAATGTGTCCTTAAACCGGTTGAATCTTCAGATATGAAAATGACTCAGCTATTCAC 1264
            ************************************************************

WT          CAAAGTTGAATCAGAAGATACAAGTAGCCTCTTTGACAAACTTAAGAAGGAACCTGATGC 1380
XL1E11      CAAAGTTGAATCAGAAGATACAAGTAGCCTCTTTGACAAACTTAAGAAGGAACCTGATGC 1324
            ************************************************************

WT          TTTAACTTTGCTGGCCCCAGCCGCTGGAGACACAATCATATCTTTAGATTTTGGCAGCAA 1440
XL1E11      TTTAACTTTGCTGGCCCCAGCCGCTGGAGACACAATCATATCTTTAGATTTTGGCAGCAA 1384
            ************************************************************

WT          CGACACAGAAACTGATGACCAGCAACTTGAGGAAGTACCATTATATAATGATGTAATGCT 1500
XL1E11      CGACACAGAAACTGATGACCAGCAACTTGAGGAAGTACCATTATATAATGATGTAATGCT 1444
            ************************************************************

WT          CCCCTCACCCAACGAAAAATTACAGAATATAAATTTGGCAATGTCTCCATTACCCACCGC 1560
XL1E11      CCCCTCACCCAACGAAAAATTACAGAATATAAATTTGGCAATGTCTCCATTACCCACCGC 1504
            ************************************************************

WT          TGAAACGCCAAAGCCACTTCGAAGTAGTGCTGACCCTGCACTCAATCAAGAAGTTGCATT 1620
XL1E11      TGAAACGCCAAAGCCACTTCGAAGTAGTGCTGACCCTGCACTCAATCAAGAAGTTGCATT 1564
            ************************************************************

WT          AAAATTAGAACCAAATCCAGAGTCACTGGAACTTTCTTTTACCATGCCCCAGATTCAGGA 1680
XL1E11      AAAATTAGAACCAAATCCAGAGTCACTGGAACTTTCTTTTACCATGCCCCAGATTCAGGA 1624
            ************************************************************

WT          TCAGACACCTAGTCCTTCCGATGGAAGCACTAGACAAAGTTCACCTGAGCCTAATAGTCC 1740
XL1E11      TCAGACACCTAGTCCTTCCGATGGAAGCACTAGACAAAGTTCACCTGAGCCTAATAGTCC 1684
            ************************************************************

WT          CAGTGAATATTGTTTTTATGTGGATAGTGATATGGTCAATGAATTCAAGTTGGAATTGGT 1800
XL1E11      CAGTGAATATTGTTTTTATGTGGATAGTGATATGGTCAATGAATTCAAGTTGGAATTGGT 1744
            ************************************************************

WT          AGAAAAACTTTTTGCTGAAGACACAGAAGCAAAGAACCCATTTTCTACTCAGGACACAGA 1860
XL1E11      AGAAAAACTTTTTGCTGAAGACACAGAAGCAAAGAACCCATTTTCTACTCAGGACACAGA 1804
            ************************************************************
```

```
WT        TTTAGACTTGGAGATGTTAGCTCCCTATATCCCAATGGATGATGACTTCCAGTTACGTTC 1920
XL1E11    TTTAGACTTGGAGATGTTAGCTCCCTATATCCCAATGGATGATGACTTCCAGTTACGTTC 1864
          ************************************************************

WT        CTTCGATCAGTTGTCACCATTAGAAAGCAGTTCCGCAAGCCCTGAAAGCGCAAGTCCTCA 1980
XL1E11    CTTCGATCAGTTGTCACCATTAGAAAGCAGTTCCGCAAGCCCTGAAAGCGCAAGTCCTCA 1924
          ************************************************************

WT        AAGCACAGTTACAGTATTCCAGCAGACTCAAATACAAGAACCTACTGCTAATGCCACCAC 2040
XL1E11    AAGCACAGTTACAGTATTCCAGCAGACTCAAATACAAGAACCTACTGCTAATGCCACCAC 1984
          ************************************************************

WT        TACCACTGCCACCACTGATGAATTAAAAACAGTGACAAAAGACCGTATGGAAGACATTAA 2100
XL1E11    TACCACTGCCACCACTGATGAATTAAAAACAGTGACAAAAGACCGTATGGAAGACATTAA 2044
          ************************************************************

WT        AATATTGATTGCATCTCCATCTCCTACCCACATACATAAAGAAACTACTAGTGCCACATC 2160
XL1E11    AATATTGATTGCATCTCCATCTCCTACCCACATACATAAAGAAACTACTAGTGCCACATC 2104
          ************************************************************

WT        ATCACCATATAGAGATACTCAAAGTCGGACAGCCTCACCAAACAGAGCAGGAAAAGGAGT 2220
XL1E11    ATCACCATATAGAGATACTCAAAGTCGGACAGCCTCACCAAACAGAGCAGGAAAAGGAGT 2164
          ************************************************************

WT        CATAGAACAGACAGAAAAATCTCATCCAAGAAGCCCTAACGTGTTATCTGTCGCTTTGAG 2280
XL1E11    CATAGAACAGACAGAAAAATCTCATCCAAGAGAGCCTAACGTGTTATCTGTCGCTTTGAG 2224
          ******************************.. ***************************

WT        TCAAAGAACTACAGTTCCTGAGGAAGAACTAAATCCAAAGATACTAGCTTTGCAGAATGC 2340
XL1E11    TCAAAGAACTACAGTTCCTGAGGAAGAACTAAATCCAAAGATACTAGCTTTGCAGAATGC 2284
          ************************************************************

WT        TCAGAGAAAGCGAAAAATGGAACATGATGGTTCACTTTTTCAAGCAGTAGGAATTGGAAC 2400
XL1E11    TCAGAGAAAGCGAAAAATGGAACATGATGGTTCACTTTTTCAAGCAGTAGGAATTGGAAC 2344
          ************************************************************

WT        ATTATTACAGCAGCCAGACGATCATGCAGCTACTACATCACTTTCTTGGAAACGTGTAAA 2460
XL1E11    ATTATTACAGCAGCCAGACGATCATGCAGCTACTACATCACTTTCTTGGAAACGTGTAAA 2404
          ************************************************************

WT        AGGATGCAAATCTAGTGAACAGAATGGAATGGAGCAAAAGACAATTATTTTAATACCCTC 2520
XL1E11    AGGATGCAAATCTAGTGAACAGAATGGAATGGAGCAAAAGACAATTATTTTAATACCCTC 2464
          ************************************************************

WT        TGATTTAGCATGTAGACTGCTGGGGCAATCAATGGATGAAAGTGGATTACCACAGCTGAC 2580
XL1E11    TGATTTAGCATGTAGACTGCTGGGGCAATCAATGGATGAAAGTGGATTACCACAGCTGAC 2524
          ************************************************************

WT        CAGTTATGATTGTGAAGTTAATGCTCCTATACAAGGCAGCAGAAACCTACTGCAGGGTGA 2640
XL1E11    CAGTTATGATTGTGAAGTTAATGCTCCTATACAAGGCAGCAGAAACCTACTGCAGGGTGA 2584
          ************************************************************

WT        AGAATTACTCAGAGCTTTGGATCAAGTTAACTGACAATTCTGCAGATATCCATCACACTG 2700
XL1E11    AGAATTACTCAGAGCTTTGGATCAAGTTAACTGACAATTCTGCAGATATCCATCACACTG 2644
          ************************************************************

WT        GCGGCCGCT-CGAGCATGCATCTAGAGG 2727
XL1E11    GCGGCCGCTTCGA--------------- 2657
            ********* ***
```

Supplementary Fig. 5: Point mutations at serine 687 to either alanine or glutamate were introduced in HIF-1α by site-directed mutagenesis. Sequence alignment of wildtype HIF-1α (wt) with the alanine (clone XL1A1) and glutamate (clone XL1E11) mutant of serine 687 is shown. Mutated sites are indicated in red.

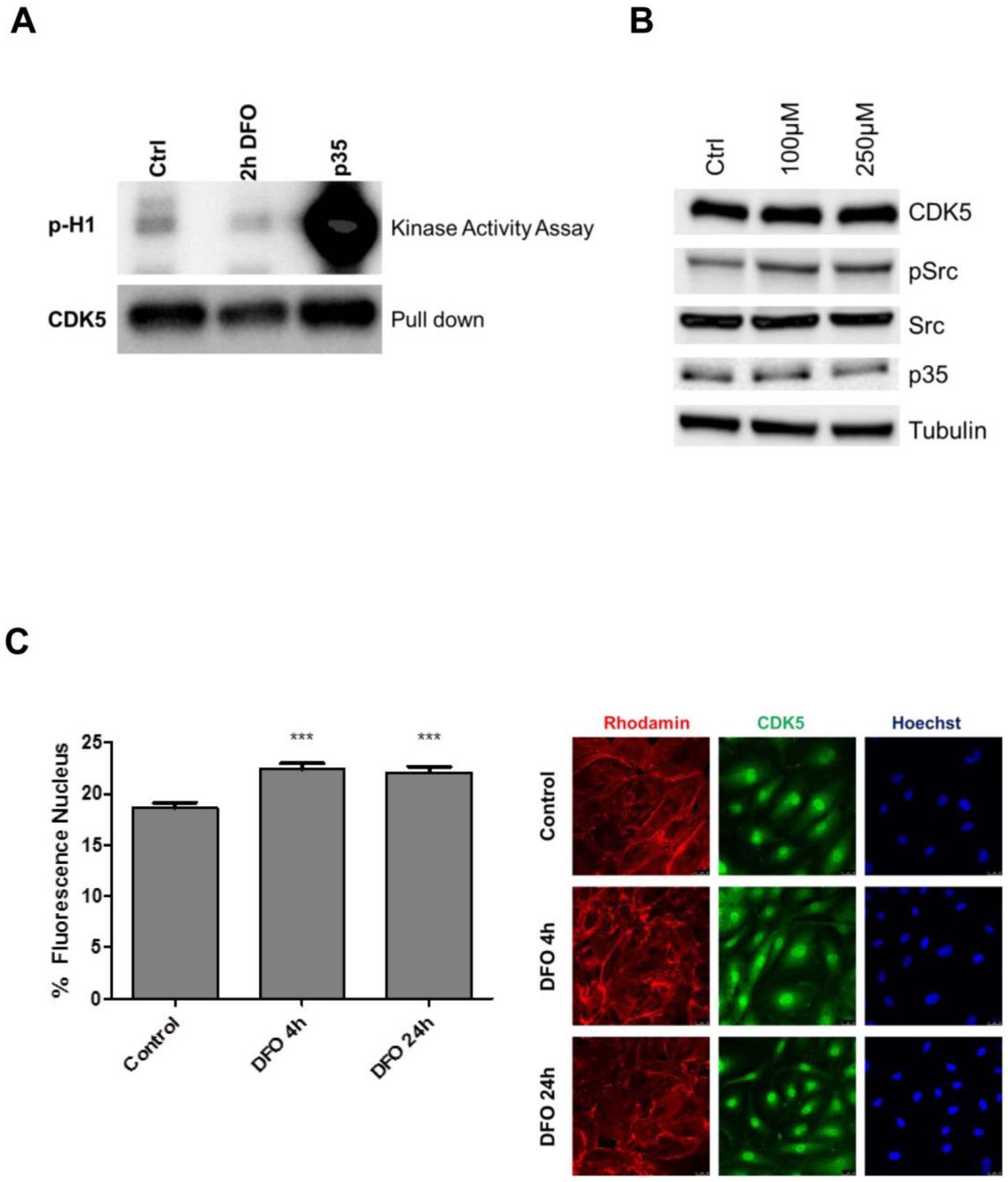

Supplementary Fig. 6: CDK5 is not directly activated by DFO treatment, but is enriched in the nucleus. (A) Kinase assay in HUVECs treated with 100 µM DFO for 2h: no increase of Histone 1 phosphorylation can be detected. Overexpression of p35 served as positice control. **(B)** Neither the CDK5 activator p35, nor phosphorylation of the downstream target pSrc is increased by DFO treatment. **(C)** Left panel: Quantitative analysis of CDK5 in the cytoplasm and the nucleus by confocal microscopy shows enrichment of CDK5 in the nucleus. Right: representative images for staining of CDK5 (green), Hoechst (blue, for identification of nuclei), and F-actin (Rhodamin phalloidin, as indicator of cell morphology).

References:

1. Ehrlich SM, Liebl J, Ardelt MA, et al. Targeting cyclin dependent kinase 5 in hepatocellular carcinoma - a novel therapeutic approach. J Hepatol 2015.
2. van den Heuvel S, Harlow E. Distinct roles for cyclin-dependent kinases in cell cycle control. Science 1993;262:2050-4.
3. Baker SJ, Markowitz S, Fearon ER, et al. Suppression of human colorectal carcinoma cell growth by wild-type p53. Science 1990;249:912-5.
4. Kondo K, Klco J, Nakamura E, et al. Inhibition of HIF is necessary for tumor suppression by the von Hippel-Lindau protein. Cancer Cell 2002;1:237-46.
